# Alcohol and Insanity

**Published:** 1910-12

**Authors:** 


ALCOHOL AND INSANITY. By Chas. L. Gregory, M. D., Superintendent of the North Texas Hospital for the Insane, Terrell, Texas. With an introduction by J. B. Gambrell, D. D., LL. D., Editor *Texas Baptist Standard*, Dallas, Texas. Cloth. Price, $1.50. Printed for the author by the Von Boeckmann-Jones Company, Austin, Texas.

This is an excellent and timely little work, now that there is on a world-wide fight against the demon, alcohol, the one great blot upon our civilization, the one great evil more far-reaching and destructive to life, health, morals and happiness than any other, the worm that is surely gnawing at the vitals of the human race, creating crime and insanity out of all proportion to increase of population. It consists of a series of twelve lectures by Dr. Gregory, delivered in several Texas cities by invitation. It deals with the effects of alcohol on the nervous system. Alcoholic Insanity, the Increase of Insanity, in relation to the nation's interests; Alcohol and Heredity; in fact, it covers the ground and presents a picture that is truly startling. I wish it could be read by every man in the world. The work is most creditable to the author, and will doubtless do much good. It matters not that Dr. Gregory quotes copiously from editorials in the "Red Back" and forgets to give credit, so that the teaching is broadly disseminated.

